# The Application of *Parmelia sulcata* Taylor and *Picea abies* (L.) H. Karst. as Biomonitors of Atmospheric Pollution in the Central Sudetes

**DOI:** 10.3390/plants15162454

**Published:** 2026-08-12

**Authors:** Katarzyna Darul, Daniel Pruchniewicz

**Affiliations:** Department of Botany and Plant Ecology, Wrocław University of Environmental and Life Sciences, pl. Grunwaldzki 24a, 50-363 Wrocław, Poland

**Keywords:** particulate matter, heavy metals, biomonitoring, lichens, coniferous

## Abstract

One of the major environmental problems in recent years is the deterioration of atmospheric air quality. Human activity in urban areas leads to the emission of particulate mat-ter containing various chemical components, including toxic heavy metals, which can accumulate in living organisms. The accumulation of pollutants by various species in mountain environments remains insufficiently understood. Therefore, the aim of this study was to assess the potential use of two species commonly co-occurring in mountain habitats—epiphytic lichen *Parmelia sulcata* Taylor and spruce *Picea abies* L., H. Karst.—as biomonitors of anthropogenic atmospheric pollution. The study was conducted in the Central Sudetes (Poland), across three site categories characterized by varying degrees of anthropogenic pressure (green, rural and urban areas). The results confirm that *Parmelia sulcata* can serve as a species reflecting heavy-metal contamination originating from atmospheric deposition, mainly lead, iron, cadmium and chromium, whereas the needles of *Picea abies* are useful mainly for indicating manganese pollution. No significant effect of anthropogenic pressure on heavy-metal concentrations was found, which is most likely related to specific air circulation in mountain areas that leads to the dispersion of air pollutants and their accumulation by the study species in the higher regions of the Central Sudetes.

## 1. Introduction

The deterioration of atmospheric air quality associated with increasing concentrations of particulate matter has become one of the most pressing environmental problems in recent years [1]. Particulate matter is considered one of the most hazardous and harmful pollutants affecting human health and life [2]. According to World Health Organization (WHO) data from 2019, as many as 6.7 million deaths annually may be associated with air pollution [3]. Particulate matter is classified according to particle size into PM_10_ (≤10 μm), PM_2.5_ (≤2.5 μm), and ultra-fine particles, i.e., PM_0.1_ (≤0.1 μm) [4]. It comprises a wide range of chemical components, including potentially toxic elements such as arsenic, cadmium, chromium, nickel, lead, copper, iron, as well as polycyclic aromatic hydrocarbons (PAHs) [5], organic compounds and biogenic volatile organic compounds (BVOCs) [6]. In urban environments, heavy metals are released into the atmosphere from various sources, such as industry, road traffic, and fossil fuel combustion [7,8].

Atmospheric air pollution can be effectively assessed using biomonitors, i.e., organisms that enable the recording of environmental changes over time [9]. Species used in biomonitoring should possess several characteristics, including high sensitivity to environmental stress, measurable responses to pollutants, and ease of observation [10,11,12]. Lichens have a number of advantages over higher plants [13] due to their high surface-to-volume ratio and efficient cation-exchange capacity [14]. Airborne pollutants are deposited on the outer layer of the lichen thallus and penetrate inward by infiltration, which is related to the absence of protective tissues or a cuticle [15]. Their long lifespan makes it possible to assess atmospheric deposition of pollutants over extended periods [16]. Among higher plants, evergreen coniferous trees are commonly used in biomonitoring of pollution in urban areas because they effectively accumulate particulate matter as a result of their thick epicuticular wax layer, needle structure, and their capacity to accumulate pollutants throughout the year [17]. No single species fully satisfies all the criteria of an ideal biomonitor for evaluating the extent of atmospheric heavy-metal contamination. It is therefore necessary to investigate different species to determine which performs best in a given biogeographical region [18]. Despite numerous studies on the bioaccumulation of pollutants by living organisms [9,10,11,12,13,14,15], there is still insufficient data to determine which taxa are best suited as biomonitors of atmospheric heavy-metal pollution in mountainous terrain. This is particularly relevant given the specific air-mass circulation associated with mountain topography.

According to data from the Chief Inspectorate of Environmental Protection, one of the localities with the highest concentrations of particulate matter is Nowa Ruda [19]. The town is located in southwestern Poland, within the Nowa Ruda Depression, which borders the Owl Mountains and the adjacent foothills [20]. The location of the town in a mountain valley limits air-mass exchange and prolongs the duration of smog episodes. Therefore, it can be treated as a suitable model area for investigating the distribution of atmospheric pollutants under conditions of varied mountain topography. The aim of the study was to evaluate the potential use of *Parmelia sulcata* Taylor and *Picea abies* L., H. Karst. as biomonitors of heavy-metal air pollution and to determine the spatial distribution of atmospheric contaminants in the Central Sudetes along a gradient reflecting difference in elevation (a.s.l.) and the intensity of anthropogenic pressure.

## 2. Results

### 2.1. Heavy-Metal Contents in the Studied Species

Heavy-metal concentrations, irrespective of the variable representing the degree of anthropogenic pressure, exhibited significant interspecific variation. In *Picea abies*, manganese concentrations were nearly three times higher compared to the concentrations found by us in *Parmelia sulcata*. Concentrations of the remaining elements were considerably higher in the lichen thalli, with the greatest differences—exceeding 30%—observed for lead, iron, cadmium, and chromium. The smallest difference (11%) was recorded only for nickel (Table 1).

### 2.2. Effect of Anthropogenic Pressure on Heavy-Metal Concentrations Parmelia sulcata and Picea abies

**Figure 1:**
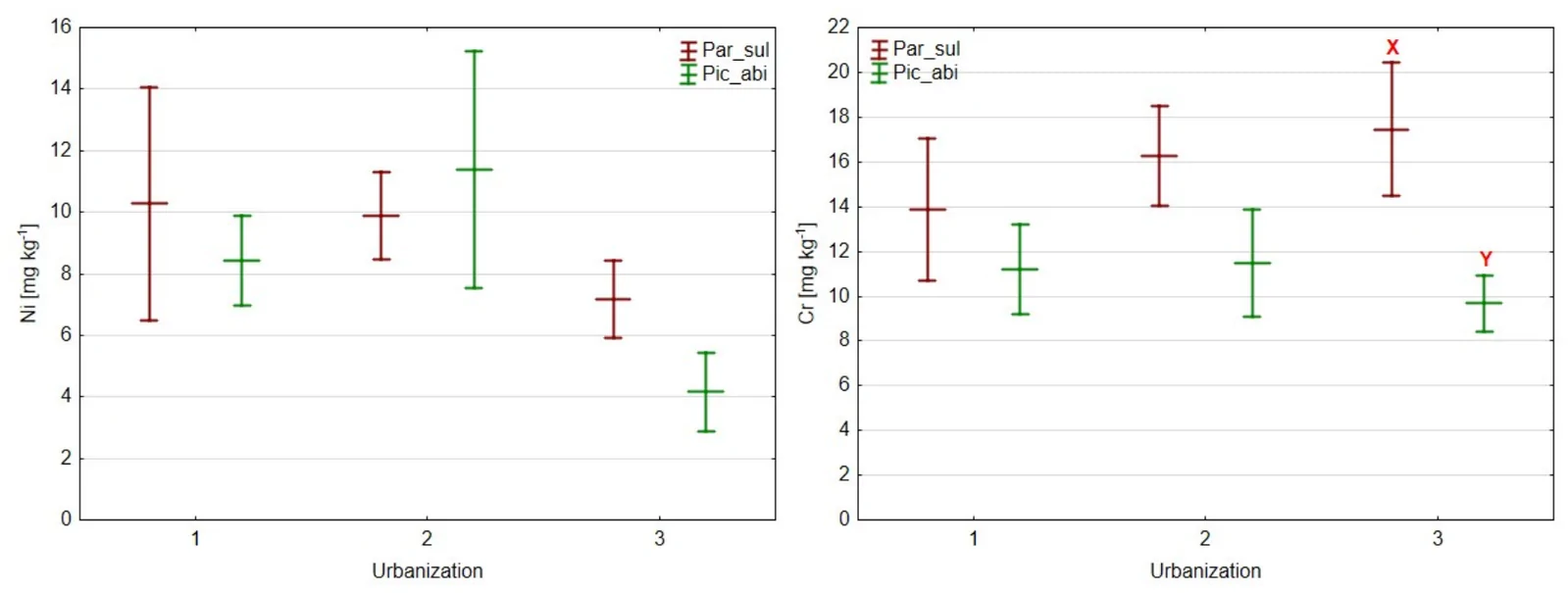

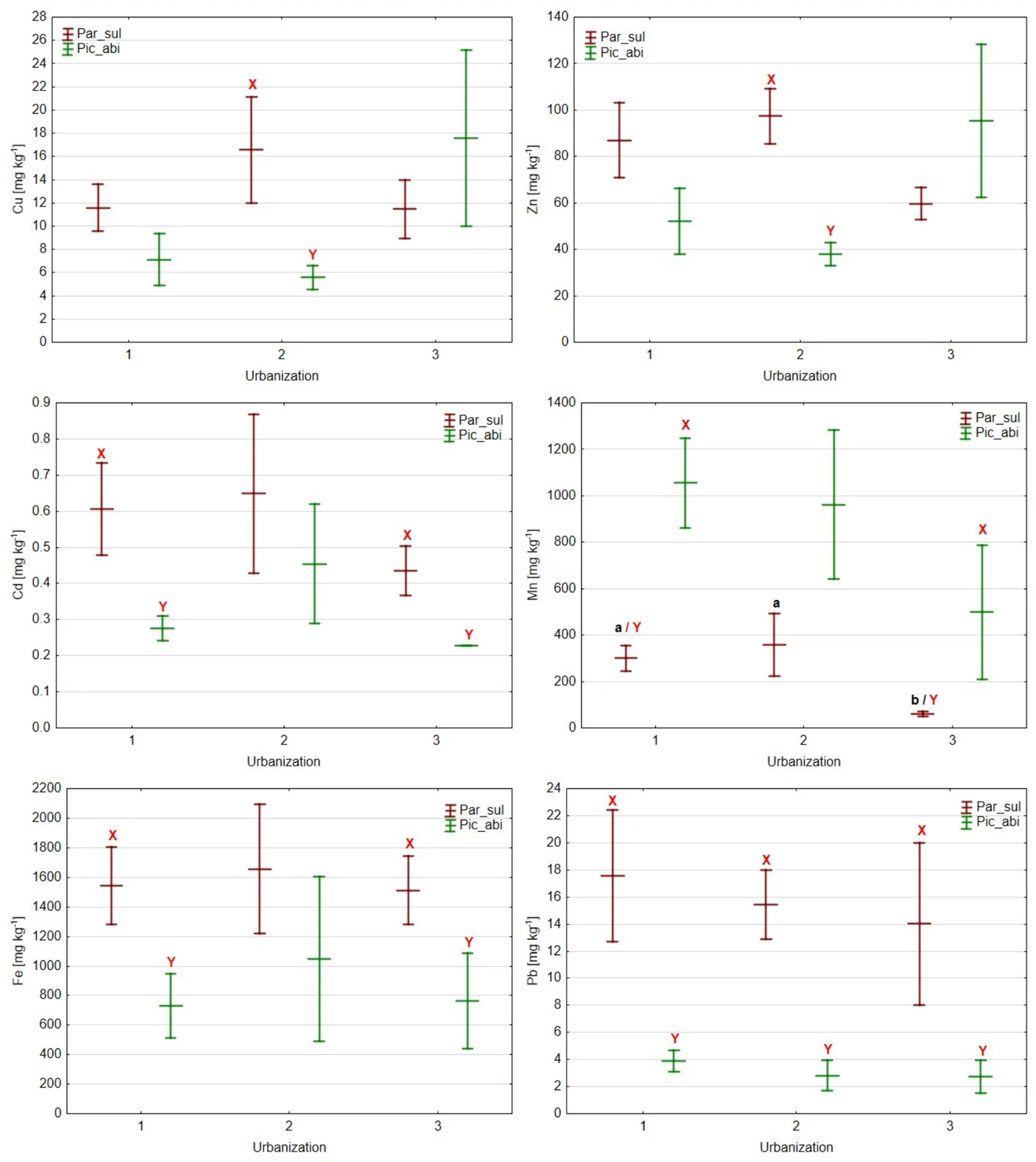
Arithmetic means with standard errors. The lowercase letters indicate homogeneous groups determined by Tukey’s HSD or Kruskal–Wallis tests among the three types of sites. Uppercase letters X and Y indicate differences between the studied species obtained using the paired Student’s *t*-test or the Mann-Whitney U test. Degree of urbanization: 1—high-elevation area without signs of anthropogenic pressure, representing “green areas” category; 2—rural areas with dispersed buildings at lower mountain elevations, representing “rural areas” category; 3—urbanized area (the town of Nowa Ruda) with the highest number of smog days, representing “urban areas” category.

Analysis of variance (ANOVA) was employed to assess the impact of anthropogenic pressure on heavy-metal concentrations in the target species, incorporating a grouping variable based on the degree of human impact: (1) high elevation areas without signs of anthropogenic pressure, representing the “green areas” category; (2) rural areas with dispersed development at lower mountain elevations representing “rural areas”; and (3) urbanized zones (the town of Nowa Ruda) characterized by the highest frequency of smog days and representing “urban areas”. Statistically significant differences were observed only for manganese in *Parmelia sulcata*, with the highest values recorded in green area plots located at higher mountain elevations and the lowest in urban areas (H = 12.247; *p* = 0.002). Comparing the accumulation of the elements between *Parmelia sulcata* and *Picea abies*, significant differences were found in green area plots for cadmium (Z = 2.578; *p* = 0.010), manganese (t = −3.731; *p* = 0.003), iron (t = 2.393; *p* = 0.034) and lead (t = 2.771; *p* = 0.017). In rural plots with dispersed buildings, significant differences in element accumulation were observed for copper (Z = 2.683; *p* = 0.007), zinc (t = 4.627; *p* = 0.001) and lead (t = 4.530; *p* = 0.001). In urban areas, significant differences were noted for chromium (t = 2.394; *p* = 0.038), cadmium (Z = 2.595; *p* = 0.009), manganese (Z = −2.322; *p* = 0.020), iron (Z = 2.002; *p* = 0.045) and lead (Z = 2.258; *p* = 0.024) (Figure 1).

### 2.3. Relationship Between Anthropogenic Pressure, Elevation, and Heavy-Metal Concentrations in Parmelia sulcata and Picea abies

**Figure 2:**
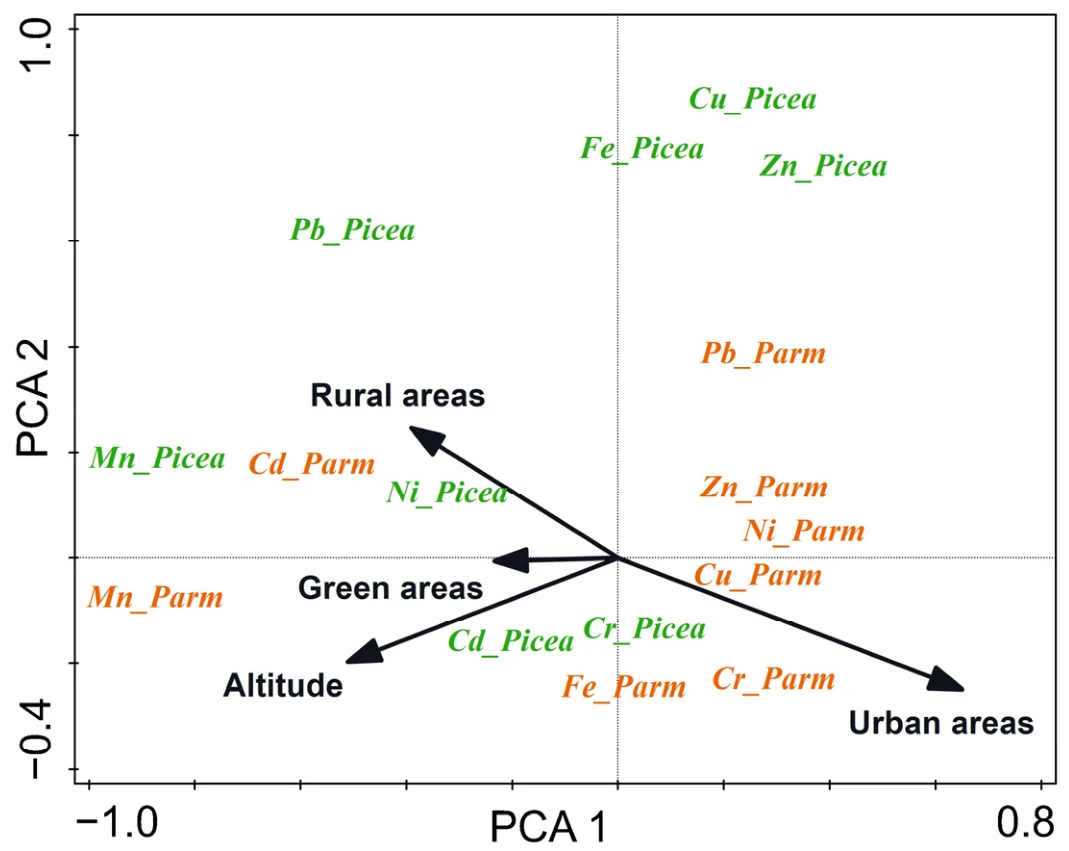
Results of principal component analysis for heavy-metal concentrations in *Parmelia sulcata* (“Parm”) and *Picea abies* (“Picea”) samples, along with variables representing anthropogenic pressure and elevation.

Principal component analysis (PCA) was conducted to visualize the distribution of pollutants along vectors representing anthropogenic pressure and elevation. Total variation was 126.825, with supplementary variables accounting for 24.59% (adjusted explained variation: 10.46%). The eigenvalues for the first and second axes were 0.2797 and 0.1957. As illustrated in Figure 2, the main gradient of variation along the first canonical axis (27.97% of explained variation) is driven by urban areas (r = 0.6503) and altitude (r = −0.5112). The second canonical axis (explaining 19.57% of the variation) is linked to urban areas (r = −0.2487) and rural areas (r = 0.2456). In the ordination space, points corresponding to chromium, copper, nickel, and zinc concentrations in *Parmelia sulcata* cluster along the urban areas vector. On the opposite side of the ordination plot, along the vectors for altitude and green and rural areas, concentrations of cadmium and manganese in *P. sulcata*, along with nickel, manganese, and cadmium in *Picea abies*, tend to concentrate.

### 2.4. Correlations in the Uptake of Individual Elements by Parmelia sulcata and Picea abies

To elucidate the potential antagonistic or synergistic relationships between the individual heavy metals recorded in *Parmelia sulcata* and *Picea abies*, nonparametric Spearman’s rank correlation analysis was employed. Analysis revealed positive associations in *Parmelia sulcata* between nickel and chromium, copper, and zinc; between chromium and lead and iron; between copper and lead and iron; between zinc and lead; and between cadmium and manganese. In *Picea abies*, positive correlations were observed between cooper and zinc and iron, and between lead and manganese (Table 2).

## 3. Discussion

Plant-based biomonitoring provides a cost-effective method for estimating air pollution levels [21]. Fungi, lichens, mosses [22,23,24], as well as tree species [8] are commonly used for this purpose. In our study, we chose to use two species that effectively capture anthropogenic pollutants: *Picea abies*, a species commonly found in mountain areas; and *Parmelia sulcata*, which frequently colonizes it. The literature highlights the fact that *Parmelia sulcata* exhibits selective and lower accumulation of heavy-metal ions compared with other lichens [25,26], even though it is widely used in European biomonitoring programs [27] and is widely distributed throughout our study area. With their lack of a waxy cuticle and stomata, combined with the accumulation and retention of heavy metals in intercellular spaces [28], lichens are highly useful for monitoring spatial patterns of trace-element deposition [29,30]. In the Sudety Mountains, Norway spruce is a common species, although it is used less frequently in research compared to another coniferous trees. In many European countries, one- and two-year-old needles of *Pinus sylvestris* and *Pinus nigra* are used to assess air pollution levels [31]. Particulate matter, which contains heavy metals, organic compounds, and biogenic volatile organic compounds (BVOCs) [6], is captured by coniferous trees, which efficiently filter pollutant particles from the atmosphere [32]. The epicuticular wax layer of needles facilitates the accumulation of lipophilic gaseous pollutants and particulate matter [33]. A fraction of these contaminants may also originate from the soil, making it difficult to distinguish between atmospheric and soil-derived pollution [34]. In our study, we focused exclusively on the one-year-old needles of Norway spruce to minimize the confounding effects of heavy-metal concentrations that could potentially originate from root translocation. The present study revealed that *Parmelia sulcata* exhibited over 30% higher heavy-metal accumulation levels compared to *Picea abies*, with the most pronounced differences observed for lead, iron, cadmium, and chromium. The opposite was observed only for manganese, for which needle concentrations in *Picea abies* were nearly three times higher. According to a study by Jurković et al. [35] on Norway spruce, higher manganese concentrations suggest a geogenic origin, which cannot be ruled out in our investigation. In our study, nickel concentrations were similar in both species, with differences around 11%. Comparing our results for heavy-metal concentrations with data from the literature [36], we found elevated levels of chromium (>10 ppm) and lead (>15 ppm) in *Parmelia sulcata*, and chromium (>10 ppm) and manganese (>850 ppm) in *Picea abies* needles. Furthermore, a comparison of the mean heavy-metal concentrations in Norway spruce needles obtained in this study with data from the literature reported for pine needles across other regions of Poland [37] reveals that our results represent higher ranges for nickel, chromium, copper (with the exception of the urbanized area of Katowice), lead and cadmium. In *Parmelia sulcata*, heavy-metal accumulation followed the order Fe > Mn > Zn > Cr > Pb > Cu > Ni > Cd, while in *Picea abies* it was Mn > Fe > Zn > Cr > Cu > Ni > Pb > Cd. Our results correspond with subtle differences reported by Kandziora-Ciupa et al. [38] for *Pinus sylvestris* needles and by Thakur et al. [39] for lichens. The observed elevated heavy-metal concentrations in lichen thalli can be attributed to their high surface-to-volume ratio and strong cation-exchange capacity [14]. This capacity results from the presence of negatively charged components in the cell wall, primarily carboxylic acid groups, which allow the formation of ionic bonds with soluble cationic elements [40], as well as the accumulation of pollutants within the thalli [15]. Additionally, tree canopies inhabited by lichens act as collectors of dry particulate matter. This material is subsequently washed off from the leaf surfaces during precipitation, and in the absence of sufficient uptake by the trees [41], it delivers pollutants directly to the lichen thalli, as was evident in our results.

The spatial separation observed in the ordination analysis reflects distinct environmental pathways of heavy-metal accumulation. Specifically, the strong association of chromium, copper, nickel, and zinc with the urban areas vector highlights the predominant influence of municipal and traffic emissions on *Parmelia sulcata*. Conversely, the clustering of cadmium and manganese in both *P. sulcata* and *Picea abies* along the altitude and green and rural areas gradients suggests either geogenic origins or the long-range atmospheric transport characteristic of higher elevations. When comparing heavy-metal accumulation between the two biomonitor species across the three groups representing different levels of anthropogenic pressure, significant differences between the species were observed in green area plots for Cd, Pb, Mn, and Fe. In rural plots with dispersed buildings, significant differences in element accumulation between *Parmelia sulcata* and *Picea abies* were found for Zn, Pb, and Cu. In urban areas, substantial differences were observed for Cr, Pb, Cd, Mn, and Fe. The economy of the town of Nowa Ruda, which represents the urban areas category in this study, is primarily focused on services and tourism. Here, the principal sources of particulate matter include solid fuel combustion in residential households and road transport along regional roads. In the case of rural areas with dispersed development, the primary pollution sources are domestic heating systems and vehicular emissions [42]. The Owl Mountains massif, where the study sites representing the green areas category were established, is devoid of local particulate matter sources. The soils in this region are characterized by a high skeletal fraction and a poorly developed soil profile. The dominant rocks in this area, primarily paragneisses and granitic gneisses with high silica content, exhibit significant resistance to weathering processes [43,44]. In the literature, heavy-metal concentrations in vegetation exhibit a decreasing trend as the distance from the emission source increases [45]. Air pollution levels are substantially elevated in urbanized zones compared to the surrounding rural areas [46,47]. The occurrence in our study of elevated heavy-metal concentrations in green areas at higher mountain elevations, which are free from anthropogenic pressure, can be explained by topography and its influence on air-mass movement. Mountain and valley winds, along with temperature inversion phenomena, affect air quality by regulating air exchange and dispersing locally generated pollutants. As a result, particulate matter and other chemical compounds can be detected in remote mountain regions far from point sources [48]. Similar results were reported by Zechmeister [49], who found a strong correlation between elevation and heavy-metal concentrations in mosses for Pb and, to a lesser extent, Cd. Similarly, Rücker and Peer [50] observed higher concentrations of Pb, Cd, and Zn in alpine regions compared with lower-elevation communities. In contrast, the study by Herman and Stefan [51] found no relationship with elevation. Kandziora-Ciupa et al. [38] reported higher Mn levels in non-polluted sites, whereas Shcherbenko et al. [52] observed a decrease in Zn concentrations with increasing pollution levels.

The data from the literature highlight the synergistic or antagonistic interactions in the accumulation of individual elements by test species. Our results confirm positive correlations in *Parmelia sulcata* between Ni and Cr/Cu/Zn; Cr and Pb/Fe; Cu and Pb/Fe; Zn and Pb; and Cd and Mn. In *Picea abies*, positive correlations were observed between Cu and Zn and Fe, and between Pb and Mn. No antagonistic relationships were detected in our study. The results for *Parmelia sulcata* partially correspond with the findings of Kord et al. [53], who reported synergistic relationships between Pb and Zn, Zn and Ni, and Cu and Ni. Kabata-Pendias and Pendias [36] note antagonism between Cr and Fe and between Cu and Fe, which was not observed in our study. Similarly, we did not detect the antagonistic relationships between Fe and Zn, Cd and Zn, or Cu and Zn described by various authors [36,52,54]. It appears that, in this case, the specific habitat conditions prevailing in the mountains may exert an additional influence.

## 4. Materials and Methods

### 4.1. Field and Laboratory Studies

**Figure 3:**
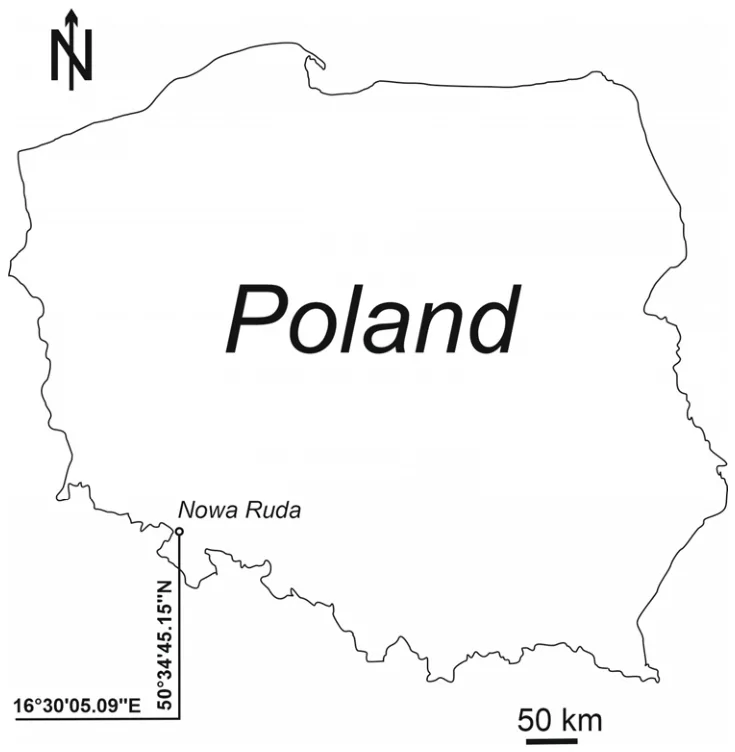
A map of the study area.

Field studies were conducted in the Polish part of the Central Sudetes (southwestern Poland) within the town of Nowa Ruda (the Nowa Ruda Depression) and the adjacent Włodzickie Hills, Wyrębińskie Hills, and the Owl Mountains massif (Figure 3). The regional nomenclature was adopted following Kondracki [20]. The elevation ranged from 374 to 822 m a.s.l. The research transect was established from west to east, following the direction of the dominant winds. Along this transect, 20 study plots representing three levels of anthropogenic pressure were designated: (1) a high-elevation area without visible anthropogenic impact, representing the “green areas” category (*n* = 7); (2) rural areas with dispersed buildings located at lower mountain elevations, representing “rural areas” (*n* = 7); and (3) an urbanized area (the town of Nowa Ruda) with the highest particulate matter pollution and representing “urban areas” (*n* = 6). At each plot, a sample of approximately 30 g of one-year-old needles of *Picea abies* (L.) H. Karst. was collected at a height of 2 m. Samples of *Parmelia sulcata* Taylor were taken in the immediate vicinity of the trees, within a maximum distance of 5 m from the sampled spruce needles. For the analysis, approximately 10 intact thalli were selected. Analogously to the spruce, lichen samples were also collected at a height of 2 m from all cardinal directions. In urban areas, *Picea abies* represented secondary plantings. Consequently, for the purposes of this study, the species was treated broadly (*sensu lato*), without distinguishing subspecies or cultivated varieties. After transport to the laboratory, the samples were gently cleaned of adhering organic debris. The samples were not rinsed prior to grinding to avoid washing away deposited particulate matter. After cleaning and homogenization, the material was dried in a forced-air oven at 80 °C until constant dry mass was obtained. Total heavy-metal contents were determined after mineralization of the plant material in a mixture of nitric and perchloric acids (4:1). Mineralization was carried out in an aluminum block at 170 °C for at least 8 h until a clear solution was obtained. Total concentrations of heavy metals were measured using flame atomic absorption spectrometry (AAS) with a Varian SpectrAA 200 spectrometer (Varian Mulgrave Victoria Australia Pty Ltd., State Victoria, Australia), following methodologies commonly applied in ecological studies [55,56]. For all elements, with the exception of chromium, background correction was performed using a deuterium lamp. Analytical grade reagents were employed throughout the analysis. During the analytical procedure, the concentrations of the target heavy metals were also determined in reagent blanks, which consisted of a nitric and perchloric acid mixture in a 4:1 ratio. The quantified trace levels of heavy metals in the blanks were averaged and subsequently subtracted from the concentrations detected in the plant tissue samples. All elements were determined using calibration standards optimized for AAS. Method validation was ensured through the analysis of a certified plant reference material. The accuracy of the reference material determination was within approximately 3% relative to its certified values.

### 4.2. Statistical Analysis

Statistical analysis of the results was performed using STATISTICA v.13 [57]. The normality of distributions was assessed with the Shapiro–Wilk test, and homogeneity of variances by the ANOVA test was evaluated using Levene’s test. Data with normally distributed values were analyzed using analysis of variance (ANOVA) with Tukey’s post hoc test, paired Student’s *t*-test, and Pearson rank correlation. Data that did not meet the assumption of normality were analyzed using nonparametric methods, including the Kruskal–Wallis test, the Mann–Whitney U test, and Spearman’s rank correlation. To assess the influence of elevation and the degree of anthropogenic pressure, principal component analysis (PCA) was conducted using Canoco v.5 [58].

## 5. Conclusions

Our results demonstrate the potential of *Parmelia sulcata* as a biomonitor of atmospheric heavy-metal pollution, particularly for Pb, Fe, Cd, and Cr, and of *Picea abies* needles for Mn. We did not observe a strong effect of anthropogenic pressure on heavy-metal uptake by the studied species. This is likely related to the specific climatic conditions in mountainous areas, which facilitate the dispersal of air pollutants and their subsequent accumulation at higher elevations that otherwise show no signs of direct human impact. To gain a deeper understanding of this phenomenon, future studies should expand their scope to include other mountainous regions and broader gradients representing varying degrees of anthropogenic pressure.

## Figures and Tables

**Table 1:** Summary of basic statistics for heavy-metal concentrations [mg kg^−1^] in *Parmelia sulcata* thalli and *Picea abies* needles.

	Mean	Standard Error	Median	Minimum	Maximum
*Parmelia sulcata*					
Nickel	9.21	1.43	7.78	1.56	30.66
Chromium	15.79	1.58	13.94	0.51	28.75
Copper	13.30	1.89	10.45	6.25	40.56
Zinc	82.43	7.78	78.41	36.36	154.77
Lead	15.74	2.50	11.93	5.68	43.18
Cadmium	0.57	0.09	0.42	0.23	1.88
Manganese	248.59	56.49	155.97	30.68	1068.12
Iron	1573.42	181.93	1570.40	482.05	3355.11
*Picea abies*					
Nickel	8.18	1.57	7.00	0.52	33.94
Chromium	10.84	1.11	10.00	4.43	23.75
Copper	9.71	2.58	5.57	2.27	52.05
Zinc	60.10	11.83	43.30	16.59	251.14
Lead	3.17	0.58	3.41	0.23	7.95
Cadmium	0.32	0.06	0.23	0.23	1.36
Manganese	855.71	158.00	540.45	78.41	2177.27
Iron	851.52	221.23	468.81	156.14	4263.98

**Table 2:** Correlations between the contents of the studied elements in *Parmelia sulcata* and *Picea abies*. Only correlation coefficients with *p* ≤ 0.05 are shown.

	Rs	t (N − 2)	*p*
*Parmelia sulcata*			
Ni–Cr	0.629	3.429	0.003
Ni–Cu	0.48	2.319	0.032
Ni–Zn	0.498	2.435	0.026
Cr–Pb	0.467	2.238	0.038
Cr–Fe	0.588	3.084	0.006
Cu–Pb	0.576	2.99	0.008
Cu–Fe	0.516	2.554	0.02
Zn–Pb	0.522	2.595	0.018
Cd–Mn	0.619	3.345	0.004
*Picea abies*			
Cu–Zn	0.556	2.84	0.011
Cu–Fe	0.53	2.651	0.016
Pb–Mn	0.478	2.307	0.033

## Data Availability

Data are contained within the article.
